# #postbabyhankypanky: An Empirically Based Knowledge Sharing Initiative About Sex and the Transition to Parenthood

**DOI:** 10.1007/s10508-020-01734-7

**Published:** 2020-06-03

**Authors:** Natalie O. Rosen, Megan D. Muise, Sarah A. Vannier, Christine T. Chambers, Heather Scott, the #postbabyhankypanky Advisory Team, Rebecca Attenborough, Kelly Chisholm, Heather Laura Clarke, James MacAulay, Marianne Pierce, Ewa Rasic, Katherine Robinson, Lisa Webb

**Affiliations:** 1 Department of Psychology and Neuroscience, Dalhousie University, 1355 Oxford Street, P.O. Box 15000, Halifax, NS B3H 4R2 Canada; 2 Department of Obstetrics and Gynecology, IWK Health Centre, Halifax, NS Canada; 3 Department of Psychology, St. Thomas University, Halifax, NS Canada; 4 Centre for Pediatric Pain Research, IWK Health Centre, Halifax, NS Canada; 5 Department of Pediatrics, Dalhousie University, Halifax, NS Canada

**Keywords:** Transition to parenthood, Postpartum sexuality, Knowledge translation, Social media

## Abstract

Despite the many sexual concerns experienced by new parents, and their reported desire for more information on this topic, both parents and healthcare providers remain reticent to broach the subject. The goal of this project was to disseminate evidence-based knowledge from our prior research in a way that was accessible, engaging, and would spark further interest and communication for both new parents and healthcare providers. We convened a multidisciplinary advisory group that also involved community parents who provided feedback at all phases of this project. We developed five brief YouTube videos, each featuring a core research finding. Following an empirically supported strategic knowledge translation plan, we disseminated the videos to our target audiences (i.e., expectant and new parents, healthcare providers, educators, and other stakeholders) using social media from February 2018 to November 2019. Data were collected using YouTube analytics and an online survey (convenience sample: *N* = 225 parents; *N* = 161 healthcare providers). From the date of the launch, the videos had a reach of 91,766 views from 14 countries, with viewers watching an average of 90% of a video. Overall, quantitative and qualitative survey results suggested that the videos were acceptable and appropriate, and respondents were more confident and comfortable discussing sexual issues (with their partner/with their patients) and would like more information about postpartum sexuality after watching the videos. YouTube videos are an acceptable and effective way to disseminate evidence aimed at raising awareness of factors affecting sexuality in the transition to parenthood.

## Introduction

Becoming a parent for the first time is typically filled with excitement and happiness, yet this transition (i.e., from pregnancy through the first year postpartum) can be fraught with significant sexual and relationship challenges for even the most committed and satisfied couples. New parents report a larger drop in relationship satisfaction compared to nonparents over the same period of time, and these declines tend to continue beyond the first year postpartum (Doss & Rhoades, 2017; Keizer & Schenk, 2012). Similar reductions in sexual function—including desire, arousal, satisfaction and frequency, and more painful intercourse—have been reported for new mothers and fathers (Maas, McDaniel, Feinberg, & Jones, 2015; McBride & Kwee, 2017). Indeed, despite the vast majority of couples (90%) having resumed sexual activity by 3 months postpartum, up to 46% of parents describe themselves as sexually dissatisfied at 6 months postpartum (Ahlborg, Dahlöf, & Hallberg, 2005). Such findings relate to the many novel sexual concerns endorsed by new parents, such as increased discrepancies in sexual desire between partners, lack of time and energy for sexual activity, changing body image, and the impact of mood on sexual activity and interest; such concerns have been endorsed by over 90% of new parents in the first year postpartum (Schlagintweit, Bailey, & Rosen, 2016). The level of sexual concerns and dissatisfaction is alarming as it can have negative implications for the individual (e.g., depressive symptoms) and the relationship (e.g., increased conflict), which in turn can have adverse consequences for the parent–child relationship, and the child’s development (Chivers, Pittini, Grigoriadis, Villegas, & Ross, 2011; Stroud, Meyers, Wilson, & Durbin, 2015; Yu, Pettit, Lansford, Dodge, & Bates, 2010).

Despite the many sexual concerns experienced by new parents, and their reported desire for more information on this topic, both parents and healthcare providers remain reticent to broach the subject. In fact, in one of the only studies that collected such data, only 18% of 480 parents reported receiving any information about sexuality postpartum, with the exception of information related to contraception and when it was safe to resume vaginal intercourse (Barrett et al., 2000). In this same study, 70% of women reported a postpartum sexual problem but only 15% of them discussed it with a healthcare provider. The narrow focus on contraception and vaginal intercourse reflects a widespread, systemic bias toward heteronormative sexuality and neglects the high diversity and psychosocial aspects of sexual relationships. Thus, despite growing knowledge in the literature regarding changes to sexuality in the transition to parenthood and its impacts, this knowledge is not being shared or used on a regular basis.

Reticence to discuss postpartum sexuality may be rooted in communication barriers between patient and provider and between romantic partners. Reported barriers include lack of knowledge and comfort with sexual topics, a focus on maternal and infant physical health during follow-up appointments to the exclusion of psychosocial or relationship well-being, societal expectations that new parenthood should be a time of joy making it difficult to raise challenges, and the fact that sexual communication between romantic partners tends to be more anxiety-provoking relative to other relationship topics leading to more avoidance (Bartellas, Crane, Daley, Bennett, & Hutchens, 2000; Guerra-Reyes, Christie, Prabhakar, & Siek, 2017; Rehman, Lizdek, Fallis, Sutherland, & Goodnight, 2017). Key consequences that may be associated with a lack of communication and knowledge about sexuality in the transition to parenthood include heightened individual and couple distress (e.g., feelings of isolation, shame) and less adaptive behaviors (e.g., avoidance, conflict) as well as missed opportunities for prevention and intervention aimed at promoting strong sexual relationships during this vulnerable time.

Taken together, there is a clear need for innovative ways to promote evidence-based knowledge of sexuality in the transition to parenthood, which may serve to normalize new parents’ experiences and open the door to conversations between partners and between parents and healthcare providers. Characteristics of the target population are essential to consider when selecting a knowledge translation (KT) strategy (LaRocca, Yost, Dobbins, Ciliska, & Butt, 2012). With the increasing popularity of parenting blogs and Web sites for both information and connecting with other parents, it is perhaps not surprising that over 75% of parents report accessing parenting information through social media (Duggan, Lenhard, Lampe, & Ellison, 2015; Dworkin, Connell, & Doty, 2013; Gibson & Hanson, 2013). In a systematic review, Moorhead et al. (2013) identified six benefits to the use of social media for health communication: increased interaction with others, more available, shared, and tailored information, increased accessibility to information, peer/social/emotional support, public health surveillance, and potential to influence health policy. Use of social media platforms such as YouTube, Twitter, Facebook, and Instagram has grown exponentially for KT efforts between researchers and their target audiences (e.g., healthcare providers, general public); social media has been shown to effectively reach both parents and healthcare providers in a manner that is accessible, relatable, and impacts clinical practice (Campbell-Yeo et al., 2017; Chambers, 2018; Lim, 2016; Maloney et al., 2015). Moreover, social media may be especially useful for engaging men, who are less likely to attend primary healthcare appointments than women (especially for mental and sexual health), although they are interested in receiving information (Robertson, 2007).

### Knowledge-to-Action Framework

The current project was rooted in the *Knowledge*-*to*-*Action (KTA) Framework* (Graham et al., 2006). The KTA framework is a guideline for the process of KT that is composed of two distinct components that are related and iterative: (1) knowledge creation and (2) the action cycle. Knowledge creation includes three components: production, synthesis, and tools/products. Regarding *knowledge production*, our research team previously conducted five cross-sectional studies with women and couples transitioning to parenthood to identify psychological factors linked to their sexual and relationship well-being. The first four studies are drawn from the same sample of 255 mixed-sex first-time parent couples^1^ who were recruited online, and the fifth study included a separate sample of 120 new mothers who were recruited from a local hospital as part of a larger longitudinal study on postpartum pain and sexuality. In both studies, participants completed validated measures online. The core findings from each of these studies are briefly summarized in Table 1; further details can be found in the published papers (Muise, Rosen, Kim, & Impett, 2017; Rosen, Bailey, & Muise, 2017; Rosen, Mooney, & Muise, 2016; Schlagintweit et al., 2016; Vannier, Adare, & Rosen, 2018). Although we and others have reviewed findings related to sexuality in the transition to parenthood (i.e., *knowledge synthesis*; Johnson, 2011; McBride & Kwee, 2017; Rosen & Byers, in press), the purpose of the current initiative was to share core findings from the aforementioned studies. Based on evidence supporting the use of social media with our target audiences, and following an empirically supported strategic KT planning tool (Barwick, 2008, 2013, 2019), we elected to create brief YouTube videos as our *knowledge products*. This process is described in more detail in the Method section.

**Table 1:** Summary of core empirical findings informing the #postbabyhankypanky YouTube videos and number of views per video

Video	Citation	Core message	Views
Video 1 *Sexual concerns*	Schlagintweit et al. (2016)	Among 239 new parent couples, nearly 90% of both mothers and fathers endorsed more than ten (of 20) sexual concerns. They rated these concerns as moderately distressing. Many sexual concerns were consistent for both mothers and fathers, while the frequency and severity of other sexual concerns differed by gender. Lower frequency and severity of sexual concerns were linked to greater relationship satisfaction of both partners	18,507
Video 2 *Seeing the other side*	Rosen et al. (2016)	When new mothers and fathers (*N* = 255 couples) reported greater dyadic empathy—a combination of perspective taking and empathic concern for one’s romantic partner—both they and their partners reported higher sexual satisfaction and relationship satisfaction	19,228
Video 3 *Differences in sexual interest*	Rosen et al. (2017)	Among 255 new parent couples, greater discrepancies in sexual desire between partners were associated with lower sexual satisfaction. However, parents felt more satisfied when fathers were the higher desire partner compared to when mothers where the higher desire partner	17,043
Video 4 *Understanding sexual needs*	Muise et al. (2017)	Higher sexual communal strength—the motivation to be responsive to a partner’s sexual needs—when that need is to have sex and when that need is to not have sex were uniquely associated with greater sexual and relationship satisfaction among 255 new parent couples. It was more important for new mothers' sexual and relationship satisfaction to have a partner who was understanding of their need not to have sex	19,206
Video 5 *Explaining sexual changes*	Vannier et al. (2018)	When first-time mothers (*N* = 120) reported the cause of postpartum sexual concerns to be more stable (i.e., likely to occur again in the future), they were less sexually satisfied, and when they attributed greater responsibility for sexual concerns to their partners, they were less satisfied with their sexual and overall relationship	17,782

The second component of the KTA framework is the action cycle, which involves a range of nonsequential activities: identifying the problem, adapting knowledge to local context, assessing barriers/facilitators to knowledge use, selecting, tailoring, and implementing interventions, monitoring knowledge use, evaluating outcomes, and sustaining knowledge use. Many of the activities in the action cycle focus on the deliberate application of knowledge to change behaviors or attitudes. The objective of the current project focused on knowledge sharing rather than explicit behavior change; thus, some of the activities in the action cycle were not relevant to our goals. The relevant activities were identifying the problem (a lack of information and communication about sexuality in the transition to parenthood among parents and healthcare providers), assessing barriers (e.g., limited time of new parents), adapting knowledge to local context (i.e., collaborative process of video production and dissemination with relevant stakeholders), monitoring knowledge use and evaluating outcomes based on our KT strategic plan, and following recommendations for evaluating implementation outcomes (e.g., acceptability; Lewis et al., 2015).

### The Current Project

Grounded in the KTA framework and adhering to the steps of a strategic KT planning tool, our objective was to develop, disseminate, and evaluate a series of YouTube videos focused on empirical factors associated with sexual and relationship satisfaction in the transition to parenthood. Our overarching goal was to share evidence-based knowledge about factors that promote sexual and relationship well-being in the transition to parenthood that would stimulate interest and communication about this topic. Although we consider the transition to parenthood to include pregnancy and the first year postpartum, we believe the videos could be beneficial beyond this period (e.g., second-time parents, parents with young children). Video reach was measured using available online analytics (i.e., number of views, countries where the videos were viewed, length of watch time) captured over the 21 months since the public launch of the videos. The effectiveness of the videos was assessed via several implementation outcomes (Lewis et al., 2015) including acceptability (i.e., liking the videos), appropriateness (i.e., perceived relevance), adoption (e.g., intention to share the videos with partners/patients), and penetration (e.g., perceived attitude change such as confidence in ability to cope with sexual problems after having a baby) in a sample of parents and healthcare providers who completed an online survey.

## Method

We followed the 13 steps of Barwick’s (2008, 2013, 2019) knowledge translation planning (KT) tool to guide this project. Partnerships that include knowledge users (i.e., parents) are critical to successful KT (Chambers, 2018).^2^ The project partners included the researchers as well as the #postbabyhankypanky advisory team, which consisted of healthcare providers (family physician, midwife, obstetrician–gynecologists), policy-/decision-makers (Reproductive Care Program of Nova Scotia), KT and social media experts, and community parents.

### #postbabyhankypanky Video Development and Dissemination

Five brief (< 1 min each) YouTube videos for parents and healthcare providers were developed, each focusing on a core research finding from our studies examining psychological predictors of sexual and relationship satisfaction in the transition to parenthood. The videos are hosted on the IWK Health Centre’s YouTube Channel and accessible through our associated Web site: www.postbabyhankypanky.com. Storyboards and scripts for the videos were developed in collaboration with Accomplice Studios (Halifax, Nova Scotia). These initial drafts went through several rounds of revisions and refinement, incorporating feedback from the #postbabyhankypanky advisory team before finalization. The videos aimed to be humorous, engaging, and inclusive (e.g., nonhuman characters, no racial identity), while still reflecting the samples of the foundational research (i.e., mainly couples in mixed-sex relationships). The end of the videos included our Web site where viewers could find more information about sexuality in the transition to parenthood including: blog posts for each of the individual research studies with further details, links to professional resources for accessing supports, and an infographic containing tips for improving communication about sex for couples.

Once produced, a public launch and video release event was held on February 12, 2018, at a local community library and live video-streamed on Facebook to increase accessibility. This event included an introduction to the project, screening of the videos, panel discussion by members of our advisory team, and questions and answers with the audience on the general topic of sexuality in the transition to parenthood. The public event was attended in person by 53 community members and was watched on the live-stream by 61 people. The videos were then disseminated using a range of strategies (Web-based and social media). Specifically, (1) press releases were sent to media resulting in six media interviews including television, online, and print publications (an article by *The Canadian Press* was distributed by over 20 news outlets); (2) team members promoted the videos through their personal and affiliated organizations (e.g., e-mails sent to listservs, posting on social media accounts); (3) pitch letters with four social media images were sent to popular parenting Web sites, bloggers, and influencers with a request to post and share; (4) pocket-sized cards with the project Web site were distributed locally to healthcare providers to give to patients, and Reproductive Care Program of Nova Scotia included the cards in prenatal form packages sent to all prenatal care providers in the province; and (5) the videos were promoted through YouTube advertising campaigns and sponsored Facebook posts (total amount spent approximately $1500). Key words (e.g., prenatal, mommy, baby bump, postpartum, new dad) were used to maximize the chances of the videos being retrieved in YouTube searches. Only data from the English versions of the videos are reported here, although French-subtitled versions of the videos were also created and uploaded and have been viewed 16,051 times.

### Data Collection and Analysis

Data pertaining to the reach of the videos were collected through social media metrics via YouTube analytics, including number of views, view duration, viewing countries, traffic to the videos and devices used to watch the videos. Google and its subsidiary YouTube do not publish their algorithms for generating this information; however, YouTube analytics are frequently used to report on reach statistics for KT videos (Campbell-Yeo et al., 2017; Harrison et al., 2016). Data were obtained from the IWK Health Centre YouTube channel reported from the launch date of February 12, 2018 to November 1, 2019.

Two online surveys (one for parents, one for healthcare providers) hosted on Qualtrics Research Suite were used to evaluate outcomes. The survey questions were modeled after similar surveys from previous KT video projects (e.g., Campbell-Yeo et al., 2017; Chambers, 2018) and were piloted and revised based on feedback from the #postbabyhankypanky advisory team. The link to the surveys was available in the text description below each video on YouTube and on the project Web site where the videos could also be accessed. Participants who completed the surveys were self-selected, and there were no specific inclusion or exclusion criteria. We obtained ethics approval for the survey from the Research Ethics Board of the IWK Health Centre.

The surveys are available on the Open Science Framework (OSF) page for this project: https://osf.io/f8xry/. General demographic questions of all respondents included their age, gender, and country. The survey questions aimed to assess acceptability, appropriateness, adoption, and penetration as per recommended outcomes for evaluating knowledge translation initiatives (Lewis et al., 2015; Proctor et al., 2011). Specifically, for the parent survey, there were eight close-ended items responded to on a 5-point Likert scale. The questions asked how much they liked the video (acceptance), how relevant it was to their life (appropriateness), and six questions about how they felt *before* versus *after* watching the videos (penetration; i.e., how concerned they were about changes to their sex life after having a baby, how much they talked to their partner about their sex life after having a baby, and how confident they felt that they could cope with changes to their sex life after having a baby). They were also asked two dichotomous (yes/no) questions about whether they were interested in learning more about sexuality in the transition to parenthood after watching the video(s) and whether they would send the link to the video(s) to anyone (adoption). The healthcare provider (HCP) survey included seven close-ended items that asked how much they liked the video (acceptance), how relevant it was to their clinical practice (appropriateness), how often they currently talk to new parents about sexual changes (adoption), and four questions about how they felt *before* versus *after* watching the videos (penetration; i.e., how aware they were of common sexual concerns of new parents, how comfortable they were talking to expectant and new parents about changes to their sex lives after having a baby). They also responded to four dichotomous (yes/no) questions about whether they will talk to new parents about sex after baby, whether they are interested in learning more about this topic, whether they would share the video with other HCPs, and whether they would use the videos in their own practice (i.e., share with patients; adoption). Perceptions of change in attitudes before and after watching the videos were analyzed using paired t tests to account for the dependence in participant responses. Effect sizes are also reported.

All respondents were given the opportunity to respond to an open-ended question in the survey regarding feedback about the videos. Qualitative data were analyzed using conventional content analysis (Hsieh & Shannon, 2005), which involves grouping text into meaningful categories or themes through a process of iterative coding undertaken by two of the co-authors (N.O.R. and M.D.M.) and reviewed by a third co-author (S.A.V.). The final coding manual is also available on the OSF page for this project.

## Results

### Video Analytics

The five videos were uploaded on February 13, 2018, and, as of the 21-month post-release date (November 1, 2019) had 91,766 total views. Table 1 shows the number of views per video. The average view time was 44 s, or 90% of the video. The videos were viewed in 14 countries, with the top five viewing countries being USA (18%), India (15%), Vietnam (6%), Canada (5%), and Philippines (4%). Over half of the viewers were male (60%), and between the ages of 25–34 (68%). Traffic to the video came primarily through YouTube advertisements (87%) that were delivered worldwide and tailored to individuals viewing parenting or baby-related videos. YouTube ads were run from March 28, 2018 to June 30, 2018, and 85% of the total views were during this period. The videos were watched on a mobile phone (57%), computer (21%), or tablet (21%).

### Survey Responses

#### Acceptance and Appropriateness

Table 2 provides the demographics for the sample of parents (*N *= 225) and HCPs (*N *= 161) who completed the surveys. On a 1–5-point Likert scale (5 representing the most positive response), parents reported liking the videos (*acceptance; M *= 3.81, SD = 1.02) and that the videos were relevant to their lives (*appropriateness; M *= 4.06, SD = 1.06). Similarly, HCPs also demonstrated high acceptance and appropriateness of the videos by reporting that they liked them (*M *= 4.15, SD = .79) and that the videos were relevant to their practice (*M *= 4.06, SD = .99).

**Table 2:** Survey demographics

	Parents (*N* = 225)	HCPs (*N* = 161)
Children^a^	%	%
Pregnant	20	–
Baby (newborn—12mo)	46	–
Toddler (1–2 years)	25	–
Preschooler (3–4 years)	23	–
School age (5–12 years)	18	–
13+ years	2	–
HCP occupation		
Doula	–	6
Psychologist	–	13
Midwife	–	27
Obstetrician–Gynecologist	–	3
Family physician	–	9
Nurse	–	21
Other*	–	14
Location^b^		
Canada	63	65
USA	12	19
Other*	8	7
Sex^b^		
Female	73	84
Male	9	6
Other	1	1

*HCP* healthcare provider

*Other occupations included health educator, prenatal yoga instructor, childbirth educator, counselor, pelvic floor physiotherapist, psychology student, lactation consultant, student midwife, doula trainer. Other locations included Germany, Australia, UK, Ireland, Switzerland, France, England, and Pakistan

^a^Parents could select more than one age-group

^b^May not equal 100% as participants had the option to not answer

#### Adoption

After watching the videos, 87% of parents expressed interest in learning more about how to cope with changes to their sex lives and 42% said that they would share the videos with others (*adoption*). The majority of HCPs (84%) were also interested in learning more about how to help their patients cope with changes to their sex lives post-baby after watching the videos and that they would share the videos with other HCPs (73%) and patients (70%) and would use them in their own practice (70%). When asked how often they currently ask new parents about sexual concerns after having a baby on a 1- to 5-point Likert scale (5 representing very often), HCPs reported that they only occasionally discussed this topic (*M* = 3.33, SD = 1.34). However, 88% reported that they would talk to patients about how they are managing sexual changes in their relationship post-baby after having watched the videos.

#### Penetration

Table 3 shows the results of paired t tests to assess changes in perceived attitudes before and after watching the videos, which reflects the evaluation outcome of *penetration*. The results of these t tests indicated that parents and HCPs perceived significant changes in their attitudes when reflecting on how they felt before versus after watching the videos. Specifically, parents reported feeling less concerned about their postpartum sex lives, more confident about talking to their partner about their sex life after having a baby and more confident in their ability to cope with changes to their sex lives after watching the videos. In addition, HCPs reported greater comfort discussing sexual issues with expectant and new parents. Interestingly, HCPs perceived a significant *decrease* in their awareness of common sexual concerns of new parents after watching the videos.

**Table 3:** Perceived changes in attitudes before and after watching videos for parents and healthcare providers

	Before watching videos	After watching videos	*t*	*d*
Parents (*N* = 191)				
How concerned were you about changes to your sex life after having a baby?	3.56	2.94	8.13**	.62
How confident do you feel talking about your sex life after having a baby with your partner?	3.02	3.82	− 8.63**	− .80
How confident do you feel that you and your partner can cope with changes to your sex life after having a baby?	3.25	3.75	− 7.38**	− .51
HCPs (*N* = 145)				
How comfortable are you talking to new or expectant parents about changes to their sex lives?	3.88	4.20	− 4.32**	− .33
How aware were you of common sexual concerns of new parents and how they affect couples’ relationships?	4.05	3.70	2.66*	.35

All items are on a scale ranging from 1 to 5

*HCP* healthcare provider

**p < .001

*p < .05

### Qualitative Responses

Parents (*n* = 55; 26%) and HCPs (*n* = 63; 42%) also provided qualitative feedback at the end of the surveys. Five themes emerged in these data through our qualitative coding: general positive, more information, normalization/communication, inclusivity, and negative video style. Table 4 provides definitions and examples for each theme, as well as the frequency with which these themes emerged. About half (47%) of the responses provided by both parents and HCPs included comments that were coded into more than one theme. Overall, the most common types of feedback from parents were generally positive comments and a desire for more information about the topic. The most common types of feedback from HCPs were generally positive comments and comments about the inclusivity of the videos. Only 8% of respondents provided comments that were coded as negative video style *only* (i.e., additional negative comments were from respondents who also made positive comments). Inter-rater reliability for each of the coded themes was strong with Cohen’s $\kappa$ > .80 (Brennan & Silman, 1992).

**Table 4:** Qualitative findings from parent and healthcare provider comments

Theme	Definition	Examples	Frequency (%) in parent survey (*n* = 55 comments from parents, *n* = 72 codes)	Frequency (%) in HCP survey (*n* = 63 comments from HCPs; *n* = 106 codes)
General positive	General positive comments about the video including plans to share/use them	“Great videos!” “Awesome initiative and very well executed”	23/72 (32%)	35/106 (33%)
More information	Comments that relate to new information that viewers would have like to have seen in the videos, or wanting more information based on the video content	“I’d like to see the issue of pain addressed” “Needs more practical advice”	22/72 (31%)	15/106 (14%)
Normalization/communication	Comments that relate to the videos normalizing concerns and encouraging communication	“Good to raise awareness and not make you feel alone” “Amazing tool to start normalizing talking about sex post-baby”	3/72 (4%)	17/106 (16%)
Inclusivity	Comments in this code include both positive perceptions of inclusivity, recommendations for improving inclusivity, and comments that the videos were heteronormative	“Creatures instead of humans help keep things gender neutral” “The videos seem fairly hetero-/cis-normative”	6/72 (8%)	20/106 (19%)
Video style (negative)	Comments in this code are general negative comments about the videos	“Overall feel are a bit childish” “A little short and simplistic”	19/72 (26%)	19/106 (18%)

## Discussion

The current project bridged an important knowledge-to-action gap in sexuality during the transition to parenthood by working collaboratively with researchers, knowledge users, and decision-makers to disseminate evidence-based knowledge via five brief YouTube videos. Grounded in the knowledge to action framework (Graham et al., 2006) and following an empirically supported knowledge translation (KT) plan (Barwick, 2008, 2013, 2019), we found that the videos were an acceptable and effective way of sharing knowledge about sexuality during this vulnerable period and stimulating awareness and interest in the topic among parents and healthcare providers.

Over the 21-month period of analysis, the videos were viewed more than 91,000 times with viewers watching 90% of the videos on average, indicating that we reached a large number of people with our KT strategy. Most parents access parenting information via social media (Duggan et al., 2015), which may be even more true for sensitive topics such as sexuality. Unfortunately, media representations of new parents’ relationships—including via social media—may paint an overly positive picture and ignore potential challenges and areas of concern or distress. Further, the available content intended to support new parents’ health and well-being is not always evidence-based, and many people struggle to tell the difference (Diviani, van den Putte, Meppelink, & van Weert, 2016). The current videos shared primarily via social media may help to break down communication barriers and normalize the sexual concerns faced by most new parents.

Indeed, results of the surveys suggested that both parents and healthcare providers liked the videos and found them relevant to their lives or clinical practice. The qualitative comments under the theme *general positive*, which also captured statements about plans to share/use the videos, were endorsed by a third of parents and a third of healthcare providers. These results reinforce that the videos were deemed acceptable and appropriate. We found strong support for our metrics of adoption with parents and healthcare providers reporting a high interest in receiving more information after watching the videos and interest in sharing the videos with others. These results together with comments falling under the qualitative theme of *normalization and communication* suggest that we met our broader goal of stimulating interest and enhancing (intentions to) communicate about sexuality in the transition to parenthood. We cannot determine, however, whether watching the videos actually resulted in any behavior change, although this was not the goal of our initiative.

Parents perceived significant decreases in how concerned they were about changes to their sex life, and increases in how much they would talk to their partner about their sex life and how confident they felt that they could cope with changes to their sex life after watching the videos relative to before they had watched the videos. Healthcare providers also perceived significant increases in how comfortable they were talking to expectant and new parents about changes to their sex lives after watching the videos. Interestingly, healthcare providers perceived a decrease in their awareness of common sexual concerns of new parents after watching the videos relative to before the videos. It is possible that healthcare providers learned new information in the videos that highlighted some of their own gaps in knowledge (e.g., of the diversity and frequency of common sexual concerns). Generally, responses from both parents and healthcare providers indicated that the videos were effective in normalizing sexual concerns and increasing perceptions of self-efficacy for coping and communication related to sexuality in the transition to parenthood. Such findings are in line with other knowledge translation tools that have been found to normalize patient experiences and consequently reduce distress (Reid et al., 2017).

Three additional themes emerged from the qualitative comments shedding further light on potential reactions to the videos. Importantly, 31% of parents and 14% of healthcare providers made comments related to the theme *more information*, that is, they wanted more information on the video content or more information on a topic that was not covered in the videos (e.g., pain). As recommended when utilizing social media for KT (Harrison et al., 2016), the videos were intentionally short and focused on a key engaging message. Our average view duration was 90%, which was higher than other similar initiatives that had longer videos (Campbell-Yeo et al., 2017; Harrison et al., 2016). Additional details about the video content could be found in the blog posts on our associated Web site, but it may be that most viewers did not go to the Web site and we did not collect statistics on Web site traffic. Still, such comments underscore the importance of providing resources at the end of the videos for those interested, especially given the short nature of the current videos. We are encouraged by the comments suggesting additional topics for future videos; we agree that there are other relevant studies about this topic that could be helpful, and such comments suggest that new videos would be welcome.

Almost a fifth of healthcare provider respondents and a minority of parents (8%) made comments that fell into the general theme of *inclusivity*, which reflected both positive and negative statements. The research was based on individuals who were in mixed-sex relationships and identified as heterosexual, limiting our ability to generalize the findings and corresponding videos to sex and gender diverse people. Although viewers appreciated our use of racially neutral characters, one of whom was conceived to be gender-neutral, other respondents perceived the videos to be too hetero- and cis-normative. Identifying with the characters depicted in KT materials is essential to maximize impact (Lohan, Aventin, Oliffe, Han, & Bottorff, 2015; Reid et al., 2017). Consistent with shifts in the fields of sex and relationships research to prioritize inclusivity (Andersen & Zou, 2015), our ongoing and future research makes a concerted effort to recruit sex and gender diverse couples and our future videos will be more responsive in this respect.

Finally, 26% of parents and 18% of healthcare providers made qualitative comments that were generally *negative about the video style* (e.g., childish, superficial). It is important to note that of these respondents, the majority (76%) also provided comments that fell into one of the other themes. In other words, very few respondents (8% overall) had only negative perceptions of the videos. It is difficult, if not impossible, to create a video that appeals to all audiences. Still, our team will give careful consideration to this feedback when developing future videos. These results highlight the essential role of a multidisciplinary advisory group in knowledge translation projects. By consulting directly with HCPs and community parents at each stage of video development, we were successful in creating a product that was viewed positively by the majority of our target audience (who responded to the surveys).

### Limitations

The response rate to the surveys was very low relative to the number of overall views—though comparable to other similar initiatives (Campbell-Yeo et al., 2017; Harrison et al., 2016; Topps, Helmer, & Ellaway, 2013)—making it impossible to know how representative the survey respondents were of our target populations. Similarly, although we tailored our advertisements to our target audiences (e.g., based on recent keyword searches), we cannot know for certain whether the viewers were indeed expectant and new parents and healthcare providers. This is a broader limitation of all mass media campaigns and one that researchers should endeavor to mitigate via careful consideration of tailored dissemination strategies. Although over half of the videos were viewed by men, survey respondents identified primarily as women, limiting the generalizability of the survey results to men or sexual and gender diverse individuals. Qualitative comments were provided by a further fraction of respondents suggesting that these are not necessarily representative of all survey respondents or the broader viewership. In general, survey respondents likely reflected people with a particular interest in the topic and who had strong opinions after watching the videos. In addition, respondents answered all survey questions at the same time (i.e., after watching the videos); thus, responses reflected perceived changes in attitudes and are not a true pre/posttest of attitude or behavior change.

Our evaluation of effectiveness focused on acceptability, appropriateness, adoption, and penetration (Lewis et al., 2015; Proctor et al., 2011). Other important implementation outcomes include feasibility (extent to which an innovation can be successfully used in a given setting), fidelity (degree to which an innovation was implemented as intended), implementation cost, and sustainability (extent to which a new innovation is maintained or institutionalized in a setting; Proctor et al., 2011). Social media campaigns may be better suited for raising awareness and influencing beliefs and motives, rather than directly changing behaviors (Boles, Adams, Gredler, & Manhas, 2014), although much more research is needed before drawing such conclusions. Thus, the additional implementation outcomes would be more appropriate should the videos be integrated into a broader intervention effort to improve sexual and relationship well-being in the transition to parenthood, which must be tested in a formal implementation trial.

### Conclusions

The wide reach of the videos and the positive response of viewers based on the survey findings suggest that the #postbabyhankypanky YouTube videos were an effective means of knowledge translation for sex research. Specifically, our results indicated that the videos successfully raised awareness and interest in evidence-based knowledge about sexuality in the transition to parenthood. Following a strategic KT plan that included partnerships with knowledge users and other stakeholders ensured that the videos had a wide appeal and reach. Still, researchers and implementation scientists must continue to work toward improving metrics for assessing the effectiveness of online campaigns using social media for KT (Maloney et al., 2015; Moorhead et al., 2013). It might be useful to examine social media products in more controlled environments first, such as in a randomized controlled trial (RCT), to establish whether they result in attitude or behavior changes before embarking on a broader mass media campaign.

In the transition to parenthood, the majority of parents experience novel sexual concerns that are often distressing and unexpected due to the limited information they have received to prepare them for these changes (Guerra-Reyes et al., 2017; Schlagintweit et al., 2016). The current evidence-based initiative of brief YouTube videos raised awareness among parents and healthcare providers about sexuality in the transition to parenthood, potentially paving the way toward improved communication and reducing distress during this joyful yet challenging life stage. There are few examples of this kind of KT initiative in sex research; the current project can serve as one model for sex researchers interested in sharing their research more widely using videos and/or social media.

## Footnotes

1 Minor deviations in sample sizes across these studies relate to incomplete measures for particular study variables, which differed depending on the study.

2 The KT planning tool can be found here: http://melaniebarwick.com/knowledge-translation-tools/.
